# New Appliances and Things Medical

**Published:** 1896-03-21

**Authors:** 


### NEW APPLIANCES AND THINGS MEDICAL.

[We shall be glad to receive, at our Office, 428, Strand, London, W.C., from the manufacturers, specimens of all new preparations and appliances, which may be brought out from time to time.]

### ITALIAN WINES.

(Egidio Vitali, 5 and 6, Great Winchester Street, E.C.)

We have received several samples of wine by the above firm, namely, Burgundy, Claret, Chianti, and Sparkling wine. Our analyst's examination of these wines has shown that they are of genuine character, and they can be recommended for general consumption, as they are agreeable in flavour. They possess, also, the additional advantages of being eminently suited for invalids, and of being procurable at very reasonable prices; for instance, the Burgundy, which is a fine rich wine, may be purchased in quart flagons at 24s. per dozen; the claret, a thoroughly sound wine for every-day use (Valtillina ordinaire), in quarts, at 16s. per dozen; and the Chianti, a red Tuscany wine, in quarts, at 18s. per dozen. As for the sparkling wine (Egidio Vitali), it is a thoroughly dry white wine, and a splendid example of what Tuscany can produce in this line. For invalid purposes we can recommend it as a substitute for champagne, unless of the very best brands or of the driest variety. At 66s. per dozen quarts, the price of this wine, it is, however, difficult to find a reliable champagne. The analytical figures for two samples of Burgundy and Chianti are given below in tabular form, so that those of our readers who are interested in the variations of wines of this character may compare the results:—

| | Chianti. | Italian Burgundy. |
|---|---|---|
| Alcohol ... ... ... | 9·79 | 9 65 |
| Total extract ... ... | 2·37 | 3·47 |
| Mineral matter ... ... | 0·23 | 0·33 |
| Total acidity in c.c. N/10 Soda per 100c.c. ... | 82 | 104 |
| Volatile acidity... ... | 28 | 27·5 |

It is thus evident that both wines have about the same percentage of alcohol, or, in other words, have had the fermentation process pushed to the same extent. The Burgundy, however, is of much fuller body, as shown by the larger amount of extract and ash. Both wines are markedly acid, and have approximately the same quantity of acetic acid present, whilst the Burgundy is characterised by the higher percentage of tartaric or non-volatile acids it contains. We fully expect that when better known to the public and the profession these wines will largely replace the more expensive French, German, and Spanish wines.

